# Uterine transarterial embolization as nonsurgical management for uterine rupture following vaginal delivery: A report of two cases

**DOI:** 10.1016/j.radcr.2022.10.031

**Published:** 2022-11-12

**Authors:** Rémi Grange, Laure-Elie Digonnet, Alexandre Mayaud, Céline Chauleur, Claire Boutet, Tiphaine Raia-Barjat, Sylvain Grange

**Affiliations:** a Department of Radiology, University Hospital of Saint-Etienne, Ave Albert Raimond, 42270, Saint-Priest-en-Jarez, France; b Department of Gynecology, University Hospital of Saint-Etienne, Saint-Priest-en-Jarez, France

**Keywords:** Embolization, Hemorrhage, Coils, Uterine rupture

## Abstract

Uterine rupture (UR) is an unexpected, rare, and serious obstetrical condition, occurring in less than 0.1% of pregnancies. Complete UR is defined as a direct communication between the uterine cavity and the peritoneum due to a complete rupture of the myometrium. Here, we present 2 cases of non-surgical management of UR following vaginal delivery, which were both treated by uterine transarterial embolization (UAE). A 26-year-old woman (G0P0) was referred to the emergency ward at 35 weeks of amenorrhea to treat the rupture of membranes, in the context of twin pregnancy. A vaginal delivery was performed and blood loss exceeded 2 liters. Gelatin sponge was injected in an attempt to occlude the right uterine artery. The injection was unsuccessful. After the medical team's discussion, it was decided to definitively occlude the right uterine artery. A 37-year-old woman (G3P3) was referred for a vaginal delivery for a medical termination at 38 weeks of amenorrhea. The ultrasound revealed a left latero-uterine pelvic hematoma, suggestive of UR. Four fibered coils were used to definitively occlude the left uterine artery. Computed tomography scan showed a progressive resorption of hematoma and satisfactory enhancement of the uterine wall in the 2 cases. Transarterial embolization may allow for bleeding to stop without resorting to exploratory laparotomy, with ad-integrum restitution of the uterine wall, and thus prevent a potential hysterectomy. The findings in these 2 cases suggest that UAE should be considered if pregnant women develop UR after delivery.

## Background

Uterine rupture (UR) is an unexpected, rare, and serious obstetrical condition, occurring in less than 0.1% of pregnancies [1]. Complete UR is defined as a direct communication between the uterine cavity and the peritoneum due to a complete rupture of the myometrium. Here, we present 2 cases of non-surgical management of UR following vaginal delivery, which were both treated by uterine transarterial embolization (UAE).

**Fig. 1:**
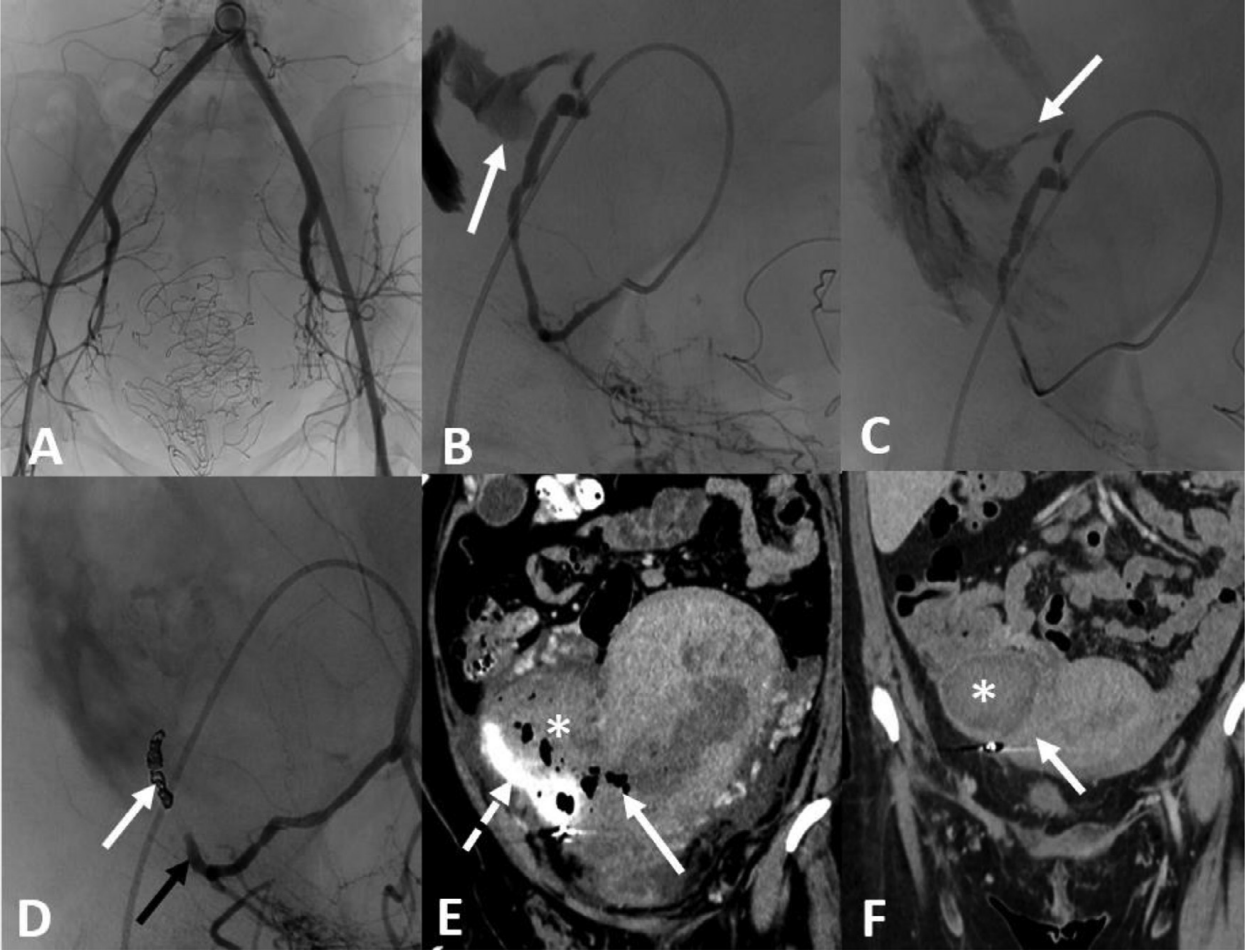
(A) Aortography after placement of 5F right femoral catheter, showing no active bleeding. (B) The selective introduction of a catheter into the right uterine artery shows active bleeding into the peritoneal cavity. (C) Angiography after injection of resorbable gelatin sponge shows slight but persistent, active bleeding. (D) After placement of the microcoils (arrow), angiography shows satisfactory occlusion of the uterine artery without active bleeding. (E) Enhanced CT scan in coronal section the day after embolization shows a gravid uterus associated with a right latero-uterine hematoma (star) with retention of contrast medium (dotted arrow), associated with a defect in enhancement and air the right uterine wall (arrow). (F) CT scan in coronal section performed 1 month after AUE shows a reduction in the size of the hematoma (star) and a restitution ad-integrum of the uterine wall.

## Case 1

A 26-year-old woman (G0P0) was referred to the emergency ward at 35 weeks of amenorrhea to treat the rupture of membranes, in the context of twin pregnancy. A vaginal delivery was performed and blood loss exceeded 2 liters. The patient was unstable, requiring blood, plasma and fibrinogen transfusion, and triple antiobiotherapy. An intrauterine tamponade balloon was placed. Transarterial embolization (TAE) was decided upon, and the patient was transferred to the operating room. After placement of a 4F introducer by right femoral approach under local anesthesia, a 4F pig tail catheter was introduced (Fig. 1). An aortography showed no active extravasation of contrast medium. The selective angiogram after introduction of a 4F Cobra catheter (Terumo, Tokyo, Japan) into the right uterine artery showed an unexpected extra-uterine leak of contrast medium, suggestive of UR. Gelatin sponge was injected in an attempt to occlude the right uterine artery. The injection was unsuccessful. After the medical team's discussion, it was decided to definitively occlude the right uterine artery after insertion of a 2.7F Progreat© microcatheter (Terumo, Tokyo, Japan), using 3 fibered coils Interlock© (Boston Scientific, Natick, USA). An angiogram confirmed the bleeding had stopped. After the procedure, a computed tomography (CT) scan angiogram found a 12 cm right latero-uterine hematoma, with a 4 cm UR without active bleeding. The patient was then transferred to the intensive care unit. The hemodynamic state gradually improved. She was discharged 6 days after delivery. An ultrasound check-up 8 days later showed good restoration of the uterine walls with homogeneous vascularization. One month after the TAE, a CT scan showed a progressive resorption of hematoma and satisfactory enhancement of the uterine wall. The patient did not present any clinical issues 6 months after returning home.

## Case 2

**Fig. 2:**
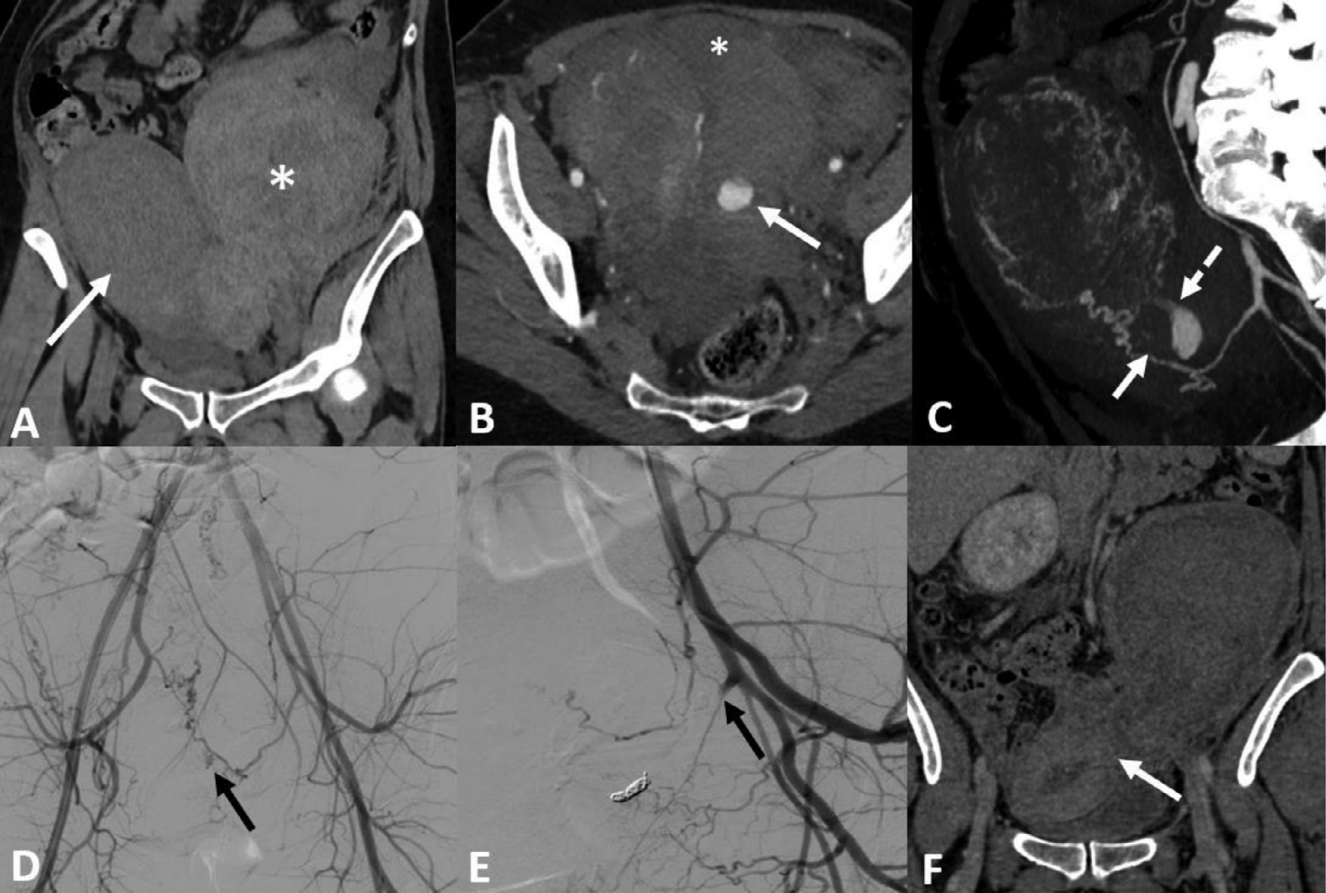
(A) CT scan without contrast injection in coronal section showing a large left lateropelvic hematoma (star) in contact with the gravid uterus (arrow). (B) CT scan with contrast injection shows active left lateropelvic hematoma (arrow) associated with hemoperitoneum (star). (C) Sagittal MIP reconstructions confirm that the bleeding (arrow) is coming from the uterine artery (dotted arrow). (D) Aortography confirms active bleeding. (E) After placement of the coils and embolization with absorbable gelatin, angiography shows satisfactory occlusion of the uterine artery at its ostium. (F) One CT scan in coronal section after injection of contrast medium shows a clear reduction in the size of the hematoma and a complete restitution of the uterine wall.

A 37-year-old woman (G3P3) was referred for a vaginal delivery for a medical termination at 38 weeks of amenorrhea for a fetal death in utero. Two hours after the delivery, the patient developed a sudden violent pelvic pain and a drop in blood pressure. The ultrasound revealed a left latero-uterine pelvic hematoma, suggestive of UR. A vascular filling was initiated, and the patient was transfused with 2 RBC units. An emergency contrasted-enhanced CT scan showed a voluminous left latero-uterine hematoma, and active bleeding in the peritoneum (Fig. 2). After a discussion between obstetricians, anesthesiologists, and interventional radiologists, UAE was decided upon. After placement of a right femoral catheter, an aortography was performed, showing no active bleeding. A fluoroscopic angiography with a Cobra Glide catheter (Terumo, Tokyo, Japan) confirmed extravasation of contrast medium from the left uterine artery into the peritoneal cavity. After placement of a 2.7F Progreat© microcatheter, 4 fibered coils Interlock© (Boston Scientific, Natick, USA) were used to definitively occlude the left uterine artery. Embolization was completed using gelatine sponge in the left and right uterine artery. An angiography showed the bleeding had stopped. The clinical status gradually improved. The next day, the patient was transferred from intensive care to a conventional hospital ward. Two days after the procedure, a CT scan confirmed the bleeding had stopped. One month after the procedure, a CT scan confirmed a decrease in the size of the hematoma. The patient returned home 3 days after the UAE.

## Discussion

The findings in these 2 cases suggest that UAE should be considered if pregnant women develop UR after delivery. UR may also cause severe morbidity and complications for the mother or the fetus [2], depending on the time between the onset of UR and medical intervention. Most of the delivery occurs before or during labor [1], resulting in emergency caesarean section. While the recommendations are consistent on the need for laparotomy exploration [3], an uterine suture or hysterectomy may be performed, depending on the extent and location of the UR. Hysterectomy after UR varies according to the retrospective studies, between 10% and 23% [1,4] in Western countries. In very rare cases, as in the 2 cases reported upon here, UR may be detected after delivery. Depending on the hemodynamic status and the possibility for interventional radiology in the structure, interventional minimally invasive management by UAE may be performed. TAE may allow for bleeding to stop without resorting to exploratory laparotomy, with ad-integrum restitution of the uterine wall, and thus prevent a potential hysterectomy. Preoperative emergency CT scans may also confirm and locate active bleeding, and anticipate UAE [5] procedures. Coils seem to be the material of choice to perform proximal and definitive UAE. In the first case reported on, resorbable gelatine sponge did not stop the bleeding due to the vascular wound. Microparticles may cause uterine ischemia by blocking distal flow and are not suitable for proximal uterine artery injury. N-butyl-cyanoacrylate allows both proximal and distal embolization but exposes patients to the risk of uterine ischemia. It is also more difficult to use.

## Conclusion

We have reported on 2 cases of non-surgical management of suspected UR after vaginal delivery, which were treated by UAE. It is necessary in these cases for good communication between the intensive care teams, obstetric surgeons, and interventional radiologists in the event that emergency surgical intervention is required.

## Consent for publication

Consent for publication was obtained for the person's data included in the study.

## Patient consent

Informed consent statement was obtained for each patient.
